# From Mice to Monkeys? Beyond Orthodox Approaches to the Ethics of Animal Model Choice

**DOI:** 10.3390/ani10010077

**Published:** 2020-01-01

**Authors:** Rebecca L. Walker, Matthias Eggel

**Affiliations:** 1 Department of Social Medicine, Department of Philosophy, and Center for Bioethics, University of North Carolina at Chapel Hill; Chapel Hill, NC 27599-7240, USA; 2 Institute of Biomedical Ethics and History of Medicine, University of Zurich; 8006 Zurich, Switzerland; matthias.eggel@ibme.uzh.ch

**Keywords:** model animals, genome editing, animal ethics, virtue ethics, primate research, translational research

## Abstract

**Simple Summary:**

New tools, allowing scientists to make precise changes to mammal genomes, have made possible future increased use of larger mammals in biomedical research, such as primates, pigs, and dogs. This paper addresses ethical issues that are raised by using larger mammals instead of smaller ones in this research. Because scientists who use animals in research follow strict guidelines, we first examine what those guidelines suggest for using larger mammals. We then consider what philosophers, who write about the ethics of animal use, consider as the important questions in evaluating which (if any) animals are acceptable to use in research. We find that philosophical perspectives have typically been interested in the question of when or if animal use is justified, while biomedical research guidance has assumed that animal use is justified but defined specific limits to that use. To address directly the ethical questions that arise in the practice of biomedical research in selecting which animals to use, we consider an approach to ethics that is focused on character and living well (or flourishing). This paper is valuable to society in drawing attention to the ethical questions, rather than merely the scientific issues, that are important in selecting which animals to use in biomedical research.

**Abstract:**

Recent developments in genome editing tools, along with limits in the translational potential of rodent models of human disease, have spurred renewed biomedical research interest in large mammals like nonhuman primates, pigs, and dogs. Such scientific developments raise ethical issues about the use of these animals in comparison with smaller mammals, such as mice and rats. To examine these ethical questions, we first consider standard (or “orthodox”) approaches, including ethics oversight within biomedical research communities, and critical theoretical reflections on animal research, including rights-based and utilitarian approaches. We argue that oversight of biomedical research offers guidance on the profession’s permitted uses of animals within a research setting and orthodox approaches to animal ethics questions when and whether animals should be used in biomedicine; however, neither approach sufficiently investigates the nuances of ethical practices within the research setting. To fill this lacuna, we consider a virtue ethical approach to the use of specific animal models in biomedicine. From this perspective, we argued that limitations on flourishing for large mammals in a research setting, as well as potential human-animal bonds, are two sources of likely ethical tensions in animal care and use in the context of larger mammals.

## 1. Introduction

If scientists can substitute the use of 10 genome-edited monkeys for the use of 1000 genome-edited mice in a search for a treatment for a devastating neurological disorder, should they do so? What if the use of monkeys offers a more promising route to such a discovery? Questions about the tradeoffs in using different types of animals as models for human disease raise important issues of relative animal moral status, comparative welfare, and the virtues of science. Yet decisions about what animal models to use in biomedicine are under-examined in the animal ethics literature, which tends to focus instead on whether and when animal research is justified. At the same time, while animal researchers are careful in selecting scientifically appropriate animal models, the ethical nuances of such tradeoffs are also left relatively unexamined by the regulatory and professional norms that guide their work. In this paper, we interrogate the ethical dimensions of potential tradeoffs between animal models of human disease, focusing, in particular, on a likely trend toward greater use of genome-edited large mammals, which could portend an eventual shift away from less successful rodent models of disease.

To examine ethical questions around such a tradeoff in the use of animals in research, we turn first to the standard (or “orthodox”) approaches, which include, on one hand, ethics oversight within biomedical research communities, and, on the other hand, critical theoretical reflections on animal research, including rights-based and utilitarian approaches. While each of these perspectives has something important to offer in considering whether and when to make tradeoffs between animal models, we identify a lacuna left open by the standard dialectic involving the mentioned approaches. Specifically, oversight of biomedical research offers guidance on the *profession’s permitted* uses of animals within a research setting and orthodox approaches to animal ethics questions *when and whether* animals should be used in biomedicine; however, neither approach investigates sufficiently the nuances of *ethical practices* within the research setting. To fill this lacuna, we consider how a virtue ethical approach to the use of specific animal models in biomedicine could add to the ethical dialogue. Before we approach these issues, we need to say more about why a shift to increased uses of larger mammals may be on the horizon for biomedicine.

## 2. Background: Scientific Rationale for a Move to Large Mammals

Since the advent of newer genome editing tools, such as editing via clustered regularly interspaced short palindromic repeats (CRISPR), over 60 ethics and policy statements regarding the use of such technologies in humans have appeared across the globe [1]. In contrast, far less attention has been given to the ethical and policy implications of nonhuman animal uses of genome editing tools despite their “immediate regulatory and scientific implications” [2]. As research animals have long been subject to genetic manipulations, it is perhaps not surprising that the use of the newer genome editing tools to create better animal models has raised little critique. Adding to the lack of public interest may be the fact that, over the past century, rodents have increasingly been the model animals of choice for genetic manipulation for translational research purposes [3]. However, the appeal of the newer genome editing tools, along with limits in the translational potential of at least some rodent models of human disease, has spurred renewed biomedical research interest in large mammals like nonhuman primates (nhps), pigs, and dogs [4].

Human clinical benefit from animal research depends on the translation of the knowledge gained in an animal model to human patients and populations. While research in mice has been the mainstay of much biomedical research progress, it has also repeatedly failed to translate into clinically relevant interventions for humans [5,6]. Reasons for this lack of translation include, but are not limited to, the mouse’s relatively short life span, small size, diet, and genomic differences to humans, each of which can lead to poor mimicking of human disease onset, development, and treatment response [7,8]. These differences, together with the low actual rate of translation of findings, help explain the increased interest in the use of animals that more closely match human biological, behavioral, and/or genomic features. While past efforts to manipulate large mammal genomes to create better models for human disease were plagued by failures, including “low efficiency, silencing, poor regulation of gene expression, and variability associated with random integration” [9] (p. 1), the newer genome editing tools, “allow those who want to understand disease processes or develop novel treatments to choose the most appropriate animal species” [4] (p. 247). According to the United States (US) Department of Agriculture statistics, for example, 2017 saw record numbers of nhps used in biomedical research generally despite increasing public opposition to such uses [10].

Human neurodegenerative conditions, such as Parkinson’s, Alzheimer’s, and Huntington’s, offer a particularly stark example of the limitations of some rodent models of human disease and illustrate why researchers may turn to nhps. Reasons for mouse models falling short in translation to clinical results in these research areas include, in addition to the issues raised above, significant developmental and functional differences between primate and rodent brains [11]. Given that human neurological and psychiatric diseases may not be fully recapitulated even when rodents are genetically modified, researchers argue “it is essential to explore genetic manipulation of non-human primates to investigate brain structures and functions and to develop in vivo models for human neurological and psychiatric diseases” [12] (p. 1). A recent request by the US National Institutes of Health (NIH) for proposals to expand marmoset colonies to promote neuroscience research is in keeping with the potential turn to nhps in this field [13]. Significantly, marmosets’ small size among primates and complex behaviors as compared with small non-primate mammals, make them a particularly appealing model animal for brain studies.

In sum, the advent of the newer genome editing tools has changed the scientific landscape in terms of the ability to genetically engineer nhp and other larger mammal models of human diseases as they give the ability to more effectively target loci in the genome and to specifically delete, and also to insert, customized DNA. While a dramatic shift in the technical ability to engineer large mammals may be tempered in reality by the costs and caretaking challenges generally associated with the use of these animals in biomedicine, it is not implausible to suggest that their use may increase rapidly in the near future. Further, such a shift may go hand in hand with a lower preference for using rodent models of human diseases, which are currently used in the millions around the world [14]. Because, in practice, fewer large mammals are used for individual protocols than are rodents (particularly mice), shifting away from the use of mice could mean using significantly fewer animals in research overall. Such a large-scale tradeoff should be examined ethically whether or not its reality comes to fruition. For ease of reference, we refer to this scenario as “the tradeoff” for the remainder of this paper.

## 3. The Animal Researcher’s Orthodoxy: Responsible Conduct of Research

Animal researchers look to professional guidance on the responsible conduct of research (RCR) when considering shifts in their practices. The RCR perspective (specifically, regulatory guidance and animal research practice norms) on the use of animals in biomedicine has largely been concerned with compliance with the 3Rs (reduction, refinement, replacement) along with an overarching principle of promoting animal welfare so far as this is compatible with science goals. This perspective creates certain boundaries around permissible uses of different animals in biomedicine; however, as we explain in this section, insufficient guidance regarding ethical issues in animal model choice that might arise in the tradeoff. We take into account US and European Union (EU) regulatory guidance as among the most developed systems of oversight available in the animal research world. That this is perceived to be the case can be seen from the heavy reliance of international accreditation services on the guidance put forth by these bodies [15].

The widely accepted 3Rs framework for animal research was initially proposed in the middle of the 20th century [16]. Since that time, it has been explicitly embraced in both the US and the EU regulatory structures [17,18]. This framework encourages the use of the least number of animals needed for the goals of the science (reduction), minimization of animal pain and distress where possible (refinement), and avoiding the use of animals where adequate non-animal alternatives exist (replacement). In addition, relative (or partial) replacement aims to substitute the use of sentient animals with animals considered to lack a similar capacity for pain and distress. What is meant, however, by “relative replacement” is a matter of some contention [19]. As originally defined, relative replacement refers to the use of animals exposed “probably or certainly, to no distress at all” [16] (p. 70). In contemporary usage, however, relative replacement has been defined in a diversity of ways, including by the widely used *Guide for the Care and Use of Laboratory Animals* as “replacing animals such as vertebrates with animals that are lower on the phylogenetic scale” [17] (p. 5). As has been pointed out by other commentators, it is unclear whether this definition refers only to replacing vertebrate with invertebrate animals (some of which are also highly sentient) or could also refer to replacing any animal higher on the phylogenetic scale with an animal lower on that scale, such as specifically the replacement of monkeys with mice [19] (p. 127). Indeed, understood in this way, something like a sliding scale of ethical consideration of animal interests may appear to overlay this phylogenetic scale [20] (pp. 35–37).

The tradeoff appears in line with reduction in so far as it offers the potential to use fewer animals in research. Such an inference accords at least with typical science practices in which fewer large animals are used per protocol than is the case with smaller animals, such as mice, although it is an open question whether statistically speaking such differences in numbers used are justified. It is important to note that, if such differences in numbers typically used are not justified, this is an ethical issue in its own right. Within the RCR approach, it is considered ethically problematic to “waste” animals either by using too few animals to achieve the scientific objectives of a study or by using more animals than those needed [21]. At the same time, using at least some larger mammals may conflict with the idea of relative replacement *if* we believe that there is a significant difference in sentience between, for example, a mouse and a monkey or generally if we interpret relative replacement expansively to encompass the broader phylogenetic scale. Since the 3Rs are not priority ranked, it isn’t clear what guidance they provide when in tension. Moreover, there are questions about how to interpret the Rs that are relevant to the scenario we suggest. For example, reduction may be understood as a norm to be applied to each research protocol in the name of efficiency or more comprehensively to the use of animals in biomedicine [22]. Also, as we have just discussed, relative replacement may refer to a phylogenetic scale or to the practice of using animals in ways that involve no pain or distress. Due to the internal conflict that is possible within the 3Rs framework, as well as differences in how the concepts are interpreted, we suggest that without further interpretation and elaboration, the 3Rs would have difficulty addressing the ethical nuances of complex decisions regarding animal model choice relevant in the tradeoff.

Beyond the 3Rs, animal research projects approved in the EU are required to meet two standards that are somewhat different than what is currently in effect in the US. In particular, they must be evaluated by a harm-benefit analysis and must not exceed an upper limit to pain and distress for the animal subjects. The harm-benefit analysis is, “to assess whether the harm to the animals in terms of suffering, pain and distress is justified by the expected outcome taking into account ethical considerations, and may ultimately benefit human beings, animals or the environment“ [18] (article 38d). While the US regulatory structure has a similar admonition that, “Procedures involving animals should be designed and performed with due consideration of their relevance to human or animal health, the advancement of knowledge, or the good of society” [23], there is no specific requirement for a harm-benefit analysis by the Institutional Animal Care and Use Committees (IACUCs) tasked with protocol approval by both the federal law (the Animal Welfare Act (AWA)) [24] and the policy covering most government-funded research (the Public Health Service Policy) [25]. Regardless, it is overall reasonable to conclude that a required harm-benefit analysis (or “due consideration” of benefit) may not offer significant ethical guidance for the tradeoff. Harm-benefit analyses are completed as assessments of individual projects and, in that way, serve as a gateway to permissible research on a case-by-case basis. Returning to our opening example of using 10 nhps instead of 1000 mice will perhaps drive the point home. As long as both the rodent and the nhp project are found to be on balance beneficial, both are permitted. While IACUCs or Animal Ethics Committees (AECs) can alternatively propose that a particular animal is not scientifically *appropriate* for use in a particular type of experiment, this is not the dynamic at issue in the tradeoff we are considering. Thus, harm-benefit analyses do not typically yield an either/or but a both/and response. This point notwithstanding, the general methods of harm-benefit analyses, either in terms of a stakeholder dialogue or predefined metrics [26], could also be utilized comparatively to assess relative harms and potential for translational value for specific model animals in light of particular scientific goals. For such comparative use of harm-benefit analyses to be relevant to protocol approval, researchers would then need to propose options of various animal models to pursue a particular scientific aim.

What constitutes an upper pain and distress limit is never made explicit in the EU Directive 2010/63. However, this issue is addressed implicitly in an annex to the Directive, which notes the permissibility of a host of interventions that are deemed to produce severe, but allowable, pain and/or distress. Examples include breeding animals with extremely dysfunctional phenotypes, inescapable electric shock, exercise to exhaustion, prolonged isolation of social species, and trauma to produce organ failure [18] (annex viii). Such a regulatory upper limit on pain and distress thus also seems to offer no helpful guidance on the issue we addressed here. The genomic modifications of larger mammals to produce models of human disease are not likely to include harm beyond these severe but allowable levels. Further, if they do so, these harms are likely to be similarly devastating for both small and large mammal species.

As a general matter, nonhuman primates and dogs are given some special mention and protection in both the US and EU oversight structures as compared to rodents, pigs, and some other species used in biomedical research. In the US, special protections include requirements within the AWA for the psychological welfare of primates and the exercise of dogs [24] (Section 2143;a;2;B). In the EU Directive 2010/63, the use of nhps is limited specifically to “those biomedical areas essential for the benefit of human beings, for which no other alternative replacement methods are yet available” [18] (preamble 17). However, a sufficient scientific rationale is perceived as justifying the use of both primates and traditional companion animal species in both US and EU biomedical research contexts. Thus, while specific obligations in the care of such animals in a research context differ from other species, and the bar might be perceived to be somewhat higher in justifying their use according to some interpretations of relative replacement and/or the sliding scale approach to weighting animal interests, such utilization is nevertheless imminently approvable.

In some ways, it is not surprising that an RCR approach offers little guidance on ethical issues arising in the choice of animal model, such as in the tradeoff. According to science’s own traditional values, animal model choice can be characterized as driven by the goals of the science and the opportunities offered by a host of practical considerations [27]. In such a context, the aim of professional ethics is to define the boundaries of permissible animal use, not to probe the ethical advantages and disadvantages of the choice of a particular animal subject. Regarding the science trend at issue, we have noted that the turn to greater usage of large animal models is motivated by potential advantages in animal to human translation, especially in some disease areas. At the same time, the animals at issue, especially primates, are very expensive to procure and to maintain, are more difficult to breed, and, because of their longer life spans, may take longer to exhibit disease states desired in the model. For these reasons a financial and rate of science efficiency point of view could argue against larger animals’ greater use. Thus, the extent to which the tradeoff takes off in biomedical research is likely to be conditioned by a balancing of these pragmatic and scientific factors—particularly in the vacuum regarding the ethics of species choice left open by the RCR framework.

## 4. The Animal Ethicist’s Orthodoxy: Rights and Utility

While animal researchers are guided by an RCR approach to their science, critical philosophical voices in animal ethics turn to another set of orthodoxies—those provided by rights-based and utilitarian moral theory. In this next section, we detail how these perspectives interrogate the presumptions of the RCR framework and where they might engage the ethics of the tradeoff. As we explain, these orthodox approaches to animal ethics attend in particular to questions of when and whether animal research is justifiable. Hence, they bring to bear specifically questions about the justifiability of the use of animals in biomedical research in evaluating the tradeoff. Thus, while RCR approaches to the tradeoff details permissible uses of animals within biomedicine, and the animal ethics orthodoxy offers critical purchase on these claims, this dialectic as a whole leaves relatively unexamined ethical questions about the very research practices involving the different types of animals at issue in the tradeoff.

### 4.1. Animal Rights: The Moral Status Objection

The tradeoff assumes that it is ethically acceptable to use mammals from mice to nhps for harmful research purposes. This assumption necessarily lies behind both the US and EU regulatory frameworks guiding animal use. Directive 2010/63 poses that this assumption is compatible with the intrinsic value of animals since it both outlines the conditions for lawful uses of animals in biomedical research and also directly affirms that animals have “intrinsic value which must be respected” [18] (preamble 12). The US animal research oversight system instead remains silent regarding the direct moral value of nonhuman animals, referring in guidance documents to the obligations of humans involved in animal research to “assume responsibility for their well-being” [17] (p. 1). Such responsibility is compatible with the animals themselves lacking direct moral value, as promoting animal welfare is also conducive to more rigorous research outcomes.

The assumptions of these regulatory frameworks notwithstanding, it may be the case that some animals have moral standing such that they have rights that would seriously restrict, or eliminate, the harm that may be done to them for research purposes. Restrictions could come, for example, through the insistence that animal rights be abrogated only in certain cases of rights conflicts. Whether or when harmful research uses of animals might constitute such cases of rights conflicts is beyond the scope of this paper. Regardless, the rights-based approach to animal ethics is so prevalent that animal protectionists may be simply conflated with promoters of animal rights by members of the general public. Even Peter Singer, a philosophical utilitarian, uses the rhetoric of animal rights in elaborating his points about protecting animal interests [28]. Nevertheless, as a philosophical approach to the modern animal ethics movement, animal rights have had a particular intellectual trajectory beginning with the articulation by Tom Regan [29]. Regan draws inspiration from Immanuel Kant’s theory about the moral status of rationally autonomous persons but argues instead that animal subjects of a life have moral status as ends in themselves. Having such a status means they should not be used as mere means to human benefit, including in biomedical research [29] (pp. 363–398). Subsequent animal rights theorists have offered different bases for animal rights, including the “political turn” in animal ethics [30,31], as well as feminist theory perspectives [32], and elaborated alternative theories of what rights are in the animals’ case (see e.g., [33]). Throughout these theoretical evolutions, the language of, and appeal to, animal rights remains a dominant orthodoxy in the animal ethics literature.

One significant way that a rights-based view would contribute to an ethical analysis of the tradeoff is if there is a moral status difference between at least some larger mammals (e.g., primates, dogs, and pigs) and at least some smaller mammals (e.g., mice) that defies the regulatory standards guiding their use. While we have already indicated how a certain understanding of relative replacement could dovetail with a sliding scale consideration of animal interests, it is also possible that those animals higher on the phylogenetic scale deserve more protections than what is offered in the current regulatory structure based on substantive features of their interests. Such a conclusion could make the tradeoff either morally impermissible or subject to certain restrictive standards. Carolyn Neuhaus, for example, argues that, while some biomedical uses of animals are acceptable, the use of genome-edited nhps to model human brain disorders is not [34]. Using David DeGrazia’s discussion of the common-sense idea of degrees of moral status, she describes that mice have an interest in avoiding pain, while humans have morally “weightier” interests in preserving their agency [34] (p. 2). She continues, “This view permits degrees of moral status ordered by the weightiness of interests. It is consistent with this view that we have some obligations to mice, for example, to avoid or at least reduce pain and suffering, but since human interests matter more, we are also justified in using mice, thereby thwarting their interests, to advance human interests” [34] (p. 2).

Does such a view offer a reason to think that nhps have rights not to be included in some harmful research that mice do not? Maybe. It is important to recognize that our obligations not to harm other individual creatures are dependent, on this view, on whether harming them furthers the interests of those with “higher” moral status (especially humans). But this kind of protection seems to fail another common sense test—of what it means for someone to have rights. In particular, it would seem a right not to be harmed should be independent of whether another set of individuals might benefit from such harm (conflicts between rights aside). Thus, while it may be true that in this picture, mice have a kind of weak moral status, they do not have rights. The question for such a view then will be whether nhps are also “below” humans on the moral status ranking according to the “weightiness” of their interests. If they are, it seems plausible to contend they also fail to have a right against being used in precisely the kinds of brain disorder research in question. Of course not having such a right doesn’t settle the issue of whether nhps ought to be so used, and Neuhaus’ argument against their use is significant in drawing on issues of animal welfare, alternatives, and lack of benefit in the face of the unsettled question about nhp moral status.

Nevertheless, it is precisely the connection between moral status and rights that we are interrogating here, and from that point of view, it seems that graduated views of moral status are on shaky ground as a basis for animal rights. As an alternative approach, the view that the moral status grounding rights is either “on” or “off” allows for moral obligation to be a special kind of attention owed on the basis of the needs and interests of individual creatures regardless of where that creature falls in a hierarchy with other creatures. There are many ethically valuable features of animals that have been addressed in the literature, including their capacity to experience pain and pleasure [35], being a ‘subject of a life’ [29], being a member of a shared society [36], or even having moral capacities [37]. The problematic implication of an on/off view of moral status, however, is that the world is divided into those who are owed moral consideration and those that are not—categories traditionally labeled “persons” and “things” where a person is a being (of any species or type) with rights, and a thing is an item that may be used as a mere means. While such a view is theoretically neater than a graduated view of moral status, practically speaking where to draw this ethical red line between creatures is highly fraught. For any basis we might select in grounding our moral consideration of animals, it is implausible that there is an in-kind difference that would support such a line being drawn between the laboratory rat and pig or, perhaps, the mouse and the monkey. Thus, it would appear that on a rights-based approach, it is plausible to suppose that *if* a monkey has a right not to be included in harmful biomedical research, then so does a mouse.

A rights-based approach to the tradeoff is thus going to be interested in the question of whether all, none, or only some of the animals being considered as models for human diseases have presumptive rights against inclusion in harmful biomedical research. This perspective interrogates the assumption of the RCR approach that any scientifically justified and socially valuable animal research is thereby permissible. The impasse in the dialectic will be if the RCR approach dictates that all such research is permitted and the rights-based approach determines none (or almost none) is ethically defensible. In such a scenario (which is not an unlikely outcome), neither approach will offer advice on ethical questions regarding actual choices between animal models taking place in research practices.

### 4.2. Animal Welfare: The Utilitarian Objection

The 3Rs offer a pragmatic framework that assumes the moral validity of animal research, and also aims to move us toward a world with less harmful, and more circumscribed, research uses of animals. However, it is important to consider what ethical principle(s) may undergird this framework. The idea of reduction, for example, assumes that it is ethically significant to use fewer, rather than more, animals to achieve a particular scientific goal. Now from a wholly practical standpoint, using more animals than needed is a waste of “resources”, including time, money, and the animals themselves. For the aim of reduction to offer an ethical goal, further reasoning is needed. The most plausible ethical argument supporting a reduction in animal use is a utilitarian objective of minimizing suffering. Similarly, for relative replacement, the assumption that it is ethically preferable to use less sentient (or non-sentient) animals most clearly relates to a utilitarian goal of an overall reduction of suffering.

If both concepts rely on a utilitarian ethical rationale, how do reduction and relative replacement withstand a utilitarian assessment and, in particular, can appeal to this moral theory offer guidance on the tradeoff that the 3Rs could not? Utilitarianism constitutes the second prong of the animal ethics orthodoxy. Although utilitarianism had been applied to animal welfare by its earliest proponent, Jeremy Bentham [38] (p. 311), the philosopher Peter Singer articulated the utilitarian perspective most associated with the modern animal protection movement. His early work argues that morality requires giving equal consideration to the interests of all sentient beings as he rejects what he terms our “speciesist” attitudes toward nonhuman animals [28] such as can also be found in a sliding scale consideration of their interests. Subsequent animal ethicists have embraced utilitarianism as an approach to animal protection (see e.g., [39,40]), and even as a way of gauging the most effective means of activism in favor of animal welfare through a movement called “effective altruism” [41].

Utilitarian metrics suggest that we maximize overall benefit (greatest welfare for the greatest number) and minimize overall harm (least harm for the fewest individuals) for all those affected by our actions (or policies). From the point of view of lowering the amount of suffering a study “costs,” the numbers of animals used is a helpful metric only if we assume that pain and distress are held steady. The ethical justification for relative replacement, however, must be that capacities for pain and/or distress are not held constant between certain species of animals. The justification for selecting mice over primates as a preferred model would thus rest on the assumption that primates, when subjected to similar experimental interventions, will suffer more. This could be the case for a number of different reasons, including a psychological capacity for more intensive suffering, a higher cognizance of pain or distress as persisting over time, or a greater concern for the suffering of conspecifics. At the same time, it may be argued that we are on thin ice when it comes to making comparative sentience claims across vertebrate species. It is unclear, for example, whether more complex intellectual or psychological capacities are associated with greater or lesser pain or distress, given similarly harmful interventions. For example, capacities that seem to promote a beings’ sensitivity to suffering may also help to ameliorate that suffering by providing sources of adaptation and resilience [42,43]. In keeping with this point, Russell and Burch’s original concept of relative replacement, as also cited above, specified that such replacement involves animals not subject to pain or distress in the actual experiment because of being used in a terminal sedation experiment, being humanely killed and used as cells, or as invertebrate or decerebrate animals incapable of experiencing pain [44]. Thus the use of a sliding scale of consideration of animal interests that overlays a phylogenetic scale, but does not reflect sentience differences, appears prejudicial from a utilitarian perspective.

Given these considerations, how would a utilitarian analysis guide us in the tradeoff? On the harm (disutility) side, a utilitarian will prefer studies with lower numbers of animals used unless an increase in harm to the animals in question leads to greater overall harm. As already noted, this issue of relative harm, in particular between species with different capabilities, is complex and difficult to resolve. On the benefit (utility) side, it will matter a great deal how effectively the studies at issue can impact human health outcomes. For this reason, the greater translation promised for research using larger mammals is a critical point for the utilitarian analysis. Accordingly, it may seem that a utilitarian will argue that the tradeoff is justified.

What this analysis fails to address, however, is unlike a 3Rs proponent, a utilitarian will not presuppose the ethical justification of the animal research enterprise. Peter Singer, for example, claims that very little biomedical research is justifiable under a principle of equal consideration of interests [35] (p. 92). The EU Directive 2010/63, by contrast, requires that research uses of animals offer a positive harm-benefit profile (a utilitarian idea), yet the implication is that such analyses are compatible with a vast biomedical research industry. While both the philosophical and RCR approach may rely on fundamentally utilitarian reasoning, then, they start from different basic assumptions about the overarching welfare benefits of animal research as well as, potentially, about the relative ethical significance of harms and benefits to humans and to other species.

Thus, the implications of a broader utilitarian ethic for the tradeoff depends both on how sanguine we are about the benefits of animal research, and how egalitarian we are about considering harms to species other than humans. It *could* turn out, for example, that while much mouse research aimed at neurodegenerative disease amelioration was *never* justifiable on a species neutral harm-benefit balancing, some primate research aimed at a similar goal is justified—if it truly has transformative effects on human health and only a small number of nhps are used. Importantly, on a species neutral balancing of benefits and harms, it could also turn out that if a small number of *humans* can be used to offer interventions that will vastly improve the quality of life of nhps, then *that too* would be justifiable.

## 5. Character and Animal Flourishing

So far, we have offered a sketch of how animal rights and utilitarian approaches to the tradeoff engage with the RCR approach by interrogating presumptions about whether and when animal research is itself justified. Yet while the resulting dialectic is illuminating regarding some of the justificatory issues raised by the tradeoff, it fails to sufficiently address ethical issues of animal model choice arising within animal research practices. A virtue-based approach helps to fill out the ethical analysis by bringing to bear character implications for each type of research, as well as a focus on the flourishing of the animals in question [45,46].

Flourishing does not simply involve avoiding pain and suffering and/or experiencing pleasure or interest satisfaction but is associated with kind-specific norms for different animals [46]. Within an Aristotelian tradition, humans are capable of *Eudaimonia*—a particular type of flourishing constituted by virtuous activity but also supported by other goods, such as friendship, family, sufficient wealth, and good health, throughout an entire lifetime. In Aristotle’s metaphysical framework, *Eudaimonia* is our natural end, or *telos*, as humans. Bernard Rollin has utilized the idea of telos as a common-sense biological concept to underwrite an ethical perspective on treating animals in ways that promote their individual natures [47]. Still, a teleological view of nature is highly contentious as a metaphysics, and it is possible to consider flourishing for both human and nonhuman animals without relying on this particular concept. Specifically, while nonhuman animals don’t flourish in the same way as humans do because they are not (at least as traditionally viewed) capable of virtue, there will be something that it means for each type of animal to live a good life. Martha Nussbaum has developed a capabilities theory according to which attending to animal flourishing is a requirement of justice [48]. Rebecca Walker has argued that there is room within a broadly Aristotelian approach to virtue ethics to ground human obligations to directly promote animal flourishing [46]. Regardless of what approach is taken, however, it will be the case that specific human virtues, such as care and compassion, require attention to animal flourishing in the contexts in which researchers and others engage with animal subjects.

Independently of the question of research-associated harms, it is much more difficult to offer a good life for some types of animals than for others in a research setting [22]. Arguably primates and dogs, for example, are extremely hard to accommodate in research settings in ways that allow them to flourish. While we can see an acknowledgment of the limitations of the research environment for these creatures in the AWA requirement that consideration is given to primate psychological welfare and that dogs be allowed to exercise, much more than exercise for dogs and attention to psychological factors for primates is at stake. For dogs, living a good life will require extensive companionship, whether with conspecifics or others (such as humans), habituation and training, time spent outdoors in enjoying open spaces and smells, avoidance of boredom, and play [49]. For nonhuman primates, engaging in appropriate social structures, foraging for food, having access to outdoor territories, and intellectual engagement are all factors in flourishing [50,51].

While some types of research facilities—such as primate centers—will presumably do better than others at making flourishing possible for these creatures, promoting animal good lives, through means such as appropriate social housing, can be extremely challenging within biomedical research [50]. Depending on the facility, animals may be confined indoors and maybe singly housed due to social ‘incompatibility’, cross-exposure, or other concerns [52]. Stereotypic behaviors, which do not occur in the wild, have been attributed to the most frequently used nhp species in biomedical research due to various welfare undermining features of captivity [53]. There are also significant de facto impediments to mouse flourishing in a typical biomedical facility [52], so we make no claim that mice flourish in such settings while larger animals do not, only that it may be more difficult to make possible a good life for larger mammals given their size, social, and intellectual needs, and the resources and space required to do so.

A utilitarian or rights-based perspective on animal research will view the acceptability of a research project as a function of its comportment to specific moral principles; however, a virtue ethical assessment depends on compatibility with the exercise of the relevant virtues and avoidance of vice. Thus, research that is inherently cruel or that is incompatible with the compassionate treatment of animal subjects is *thereby* unethical. Research projects or practices in which the practically wise researcher would engage, however, are to be promoted *in the manner* of the virtuous person. That is, in ways that track appropriate consideration, perception, emotion, and motivation [54].

Because the individual virtues, as stable character traits, are responsive to each specific context, they carry with them ready-to-hand obligations to attend to the needs and interests of nonhuman animals at the point of engagement. To give a few examples: compassion is oriented to the alleviation of suffering, loyalty is manifested in allegiance to particular animals, and care is oriented to meeting the needs of those who are in some sense dependent on us. Intellectual virtues, such as intellectual humility, honesty, and integrity, will also be critical for good science practices, for example, accurately perceiving and portraying both the likely human (or animal) benefits and actual animal harms involved in any research project.

How do these considerations of virtue engage with an ethical assessment of the move to increased use of larger animal models in lieu of larger numbers of smaller animals? A key question is whether the practically wise animal researcher would engage in each type of research. That question, however, is too vague to give us much traction. We have already noted concerns about large animals’ ability to flourish in a biomedical research setting. To the extent that they are not able to flourish, then, care and compassion may be incompatible with their use. This analysis may mean that some research is compatible with virtue only within certain types of facilities. The importance of context-sensitivity for any virtue ethical analysis must be reinforced at this juncture, as it is neither the case that all nhps, for example, are large animals nor that the limitations on meeting their needs are similar for all species. Olsson and Sandøe point to marmosets’ comparatively small size, ease of breeding in captivity, and the potential for good housing in analyzing the ethical implications of establishing transgenic marmoset colonies [55].

Also significant, is a consideration of human-animal bonds (HABs) in a research setting. While relationships between nonhuman and human animals are not friendships in a robust Aristotelian sense (which requires, among other features, shared virtue), they create bonds between particular humans and particular other animals in which both parties are participants [56]. Because virtue ethics, at least on an Aristotelian picture, makes room for ethical partiality [57], the virtue of care in the context of HABs brings along special obligations to particular others.

HABs are typically more significant in species like primates, pigs, or dogs. This is not only because of the smaller numbers of animals with whom researchers work, but also because of the greater ability of these species to reciprocate human attention. Because these species are susceptible to HABs, such relationships are also crucial to the success of the research enterprise. For example, developing habits of compliance with research-related interventions (blood draws, etc.) lowers stress levels for the animals [58]. Such bonds, moreover, may contribute to good habits on the part of the researchers themselves who will remain attentive to animal needs, be better able to recognize the animals as individuals with their own interests, and also gain benefit from the relationship. Taking these factors into account, animal suffering may actually decrease in the use of some nhps over mice if it becomes “easier for scientists and caretakers to recognize signs of suffering and [by] increasing the human motivation to limit it” [55] (p. 181).

Yet forming relationships with research animals may also create a significant ethical tension for the use of larger animals when the research involves harming the animal subjects [59]. In short, although it seems incumbent that researchers do form such bonds, when research uses are harmful, they also imply a greater violation of a duty to care. While researchers have welfare obligations to the animals in their studies regardless of HABs, the particular tension introduced by HABs is arguably a more significant problem with respect to animals more able to bond with humans. Nevertheless, the use of large mammals may also give researchers more of an opportunity to develop virtues, such as compassion and care, as well as grapple with issues like justice, that are essential to good character. If this is indeed the case, the ethical conundrum is in how to make such developed character traits fully compatible with the uses to which such animals are put.

On a virtue ethical analysis, two lines of argument imply that research using some large mammals can be more ethically fraught than similar research using some smaller mammals. These are the limitations on flourishing for some larger animals in a research setting, and the HABs that are more likely to result in irresolvable tensions in animal care and use in the context of larger mammals. Neither argument establishes that it is impossible for the practically wise researcher to use larger mammals in biomedical research, nor do they imply that the practically wise researcher necessarily is justified in using smaller mammals. A virtue ethical approach to the tradeoff instead asks us to address the specific context of the research in question in considering whether the project can be conducted in the manner of the practically wise. Answering this question will require attending to more than whether the research is on balance beneficial or whether the types of animals involved have rights against being harmed. It will require looking at the actual capacity for individual animals to flourish in a particular research setting, at the habits of thought and action that a research environment promotes, and ultimately whether specific moral and intellectual virtues are compatible with the research program in question. To be clear, there is no particular reason why a rights-based or utilitarian approach to animal ethics is constrained against bringing considerations of human virtue and animal flourishing to bear in a contextually responsive assessment of animal research. Nevertheless, the mainstay of such approaches has been otherwise while the particular toolbox of virtue ethics readily offers these considerations. 

We have argued that while RCR delimits boundaries to permissible research and the orthodox approaches to animal ethics question these boundaries on the basis of specific moral principles, virtue ethics looks to individual research contexts and their implications for human and animal flourishing in assessing the tradeoff. In some ways, then, a virtue ethical approach to the tradeoff asks us to move from idealized moral principles regarding our treatment of animals to an investigation of real-world implications of the research enterprise for human character and animal flourishing. Charles W. Mills has claimed, “the best way of realizing the ideal is through the recognition of the importance of theorizing the *non*ideal” [60] (p. 166). Yet, virtue ethical theories are far from non-ideal—especially perfectionist versions. In the final section of this paper, we discuss the way in which ideal and non-ideal factors contribute to the moral question of the tradeoff.

## 6. Discussion: Real vs. Ideal World Considerations

There are several significant ways in which an interrelation between ideal and real-world theorizing arises for this paper. The first idealized presumption is the pretense that actual tradeoffs might take place between studies using very large numbers of rodents and studies using small numbers of larger mammals. This idealization draws attention to the fictionalized but ethically salient tradeoff. In the real world, the tradeoff itself will be simply diffused throughout the broader animal research world. We have used the fiction of the tradeoff to put a fine point on the kinds of ethical conundrums that may be involved in the trend toward the use of larger mammals. Yet, it is unlikely that individual researchers (or oversight groups) will face this conundrum as such. Moreover, while it is indubitable that large animals are used on average in much smaller numbers for individual protocols than are mice and some other smaller mammals, it is unclear how, statistically speaking, a researcher would justify a direct tradeoff of this sort. It is more likely that the move to use greater numbers of larger animal models will be revealed by shifting funding priorities or global trends in research, as is already occurring in China’s “miniature explosion of genetic engineering in monkeys” [61].

How does theorizing the non-ideal in this sense change the ethics? Significantly, we don’t actually know whether increased use of primates, pigs, and dogs will, in fact, mean less use of mice and rats. It is possible instead that the uses of all these animals will simply increase as translational hurdles for biomedical science seem to lower. It is therefore important to address concerns about potential increased suffering, moral status barriers, species neutral harm-benefit analyses, and virtue ethical factors as relevant to each particular study regardless of any specific exchange that may (or may not) take place with the use of other animals.

The second way in which ideal and non-ideal theorizing are relevant for our paper is in the ideal of the virtuous or practically wise person. In Aristotle’s framework, a practically wise person is a kind of perfect human whose exercise of any particular virtue is as part of a unified and integrated character. That person can only be called “eudaimon” at the end of his (women were not viewed as capable of full virtue) life and had to be raised in a well-ordered political system with the external supports of family and health, and even have certain physical features [62]. Prejudicial features of this view aside, the political circumstances alone required for such a person to develop may imply none of us can hope to attain this ideal. Further, our modern sensibilities have shifted our beliefs away from parts of Aristotle’s vision of the virtues. We now tend to believe both that one can have some virtues without having others and that virtues are not so stable under shifting social circumstances [63].

What is important from a non-ideal theory perspective, then, is what an appeal to virtue might get us in the way of striving toward that ideal—toward improved, if flawed, character. We have argued that the use of larger mammals in research is fraught from a virtue ethical perspective due to obligations arising out of HABs, as well as concern for animal flourishing. We have also drawn attention to the ethical importance of habits engendered in the research setting—and the need to find institutional ways to support the development of care, compassion, honesty, integrity, and intellectual humility, among other traits. These ethical factors thus require attention, even if we cannot definitively answer the question of whether individual researchers are themselves virtuous.

The third intersection of the ideal and non-ideal arises from a presumption of animal research science itself. The ideal goal of animal research should be to phase out the harmful use of animals altogether. Animal researchers themselves may promote this goal and yet see it as incrementally realizable (if at all) in the real world only through the 3Rs [64]. An important question, then, is whether a move to larger animal models is a move toward or away from the idea of phasing out harmful animal use.

One could argue that the potentially more translational use of larger animal models is a step in the right direction of realizing this goal. It is possible that both overall numbers of animals used will drop, and that the use of larger animals will lead to cures or scientific understandings of particular human diseases and conditions that will allow animals to no longer be used in these areas of research at all. Yet, as noted above, it is also plausible that animal use numbers may increase overall as greater use of larger animals is simply added to the already prevalent uses of rodents. Further, if translational science is truly improved by the use of larger animals, this may appear as an argument for ramping up such uses, rather than phasing them out.

Finally, because research on dogs and primates, in particular, may capture social imaginations in ways that research uses of mice and rats do not, there is an open question of how societies will respond to increased use of larger mammals. Such responses are also likely to be culturally and temporally dependent—a reflection of a societies’ character in a broader sense. For example, in China, there is reportedly a growing interest in protecting dogs (which were previously not objects of compassion) but less interest than in the US in avoiding harmful uses of nhps [61]. It is possible that the trend to greater uses of larger mammals will lead to increased social pressure to stop some such animal research practices [10]. Alternatively, members of the general public could become desensitized to the harmful use of typical objects of compassion, allowing for a slide into possibly cruel uses of such animals. In either case, the argument that such research is “necessary” because of the greater promise for human health could motivate acceptance of an increase in the use of genome-edited large mammals *even if* these uses are otherwise seen as ethically problematic. To tackle this issue, we need to address our collective character and what we value as a society.

## 7. Conclusions

A turn toward greater use of genome-edited large mammals in biomedical research may hold the promise of better translation to human patients and populations. At this juncture, claims along these lines are largely based on the presumption that similarities between larger mammals and humans will play out in greater applicability of animal data to human health. While the RCR framework for animal research offers significant oversight of research endeavors and promotes animal welfare and reduction, refinement, and replacement, this perspective also takes for granted the ethical permissibility of harmful research uses of animals and provides insufficient critical purchase on the potential ethical distinctions between uses of different species of mammals. Rights-based and utilitarian approaches to animal ethics bring wider moral theory to bear in questioning the ethical presumptions of the RCR framework and engage the tradeoff as a justificatory question about when and whether certain animals may be used in harmful research endeavors. The dialectic between the RCR and orthodox animal ethics views offers a perspective relevant to the tradeoff but leaves a space open for addressing additional ethical complexities in animal model choice arising within the practices of animal research. We have suggested that space may be filled by: consideration of animal flourishing (beyond mere welfare) in the particular research setting, HABs and the obligations of care, and research and institutional practices that allow for the development of virtuous habits of thought and action. While virtue ethics looks to the practically wise for moral guidance, we have suggested that we may need to be content with looking to the wise-enough.

Our thought experiment in the tradeoff is meant as a catalyst for comparative ethical analysis. Yet, more realistically, the trend toward greater use of dogs, pigs, nhps, and other large mammals is unlikely to yield specific exchanges of the type we describe. The conundrum, then, is whether this trend will indeed lead to fewer uses of animals overall due to improved efficacy of the science, or whether uses of larger mammals will increase while uses of rodents continue apace. Further complicating matters is whether increased usage of larger mammals will help to sensitize scientists and the public to the ethical difficulties and complexities of such research, or whether these uses might instead allow for a greater distancing from the plight of these traditional objects of compassion. While it is too soon to determine whether the turn to greater use of genome-edited large mammals is ethical folly, it is certainly time to take up the question.
